# Supplementary material for: Spatial aggregation choice in the era of digital and administrative surveillance data
Source: PLOS Digit Health. 2022 Jun 3;1(6):e0000039. doi: 10.1371/journal.pdig.0000039 (PMC9931313; doi:10.1371/journal.pdig.0000039)
Supplement: S1 Text — The Supporting Information includes Table A and B and Figures A to W. (PDF) [file pdig.0000039.s001.pdf]

S1 Text: Supporting Information

Contents

S1 Supplementary Methods 2

S2 Supplementary Results 3

S2.1 County population has no consistent association with onset timing . . . 3
S2.2 County population has no consistent association with peak timing . . . 7
S2.3 Influenza season features across spatial scales . . . 11
S2.4 Operational implications of spatial aggregation difference . . . 13
S2.5 Heterogeneity is associated with greater spatial aggregation differences . . . 14

Supplementary Figures

A Onset timing by population for all counties with epidemics in the 2002-2003 influenza season 3
B Onset timing by population for all counties with epidemics in the 2003-2004 influenza season 4
C Onset timing by population for all counties with epidemics in the 2004-2005 influenza season 4
D Onset timing by population for all counties with epidemics in the 2005-2006 influenza season 5
E Onset timing by population for all counties with epidemics in the 2006-2007 influenza season 5
F Onset timing by population for all counties with epidemics in the 2007-2008 influenza season 6
G Onset timing by population for all counties with epidemics in the 2008-2009 influenza season 6
H Peak timing by population for all counties with epidemics in the 2002-2003 influenza season . 7
I Peak timing by population for all counties with epidemics in the 2003-2004 influenza season . 7
J Peak timing by population for all counties with epidemics in the 2004-2005 influenza season . 8
K Peak timing by population for all counties with epidemics in the 2005-2006 influenza season . 8
L Peak timing by population for all counties with epidemics in the 2006-2007 influenza season . 9
M Peak timing by population for all counties with epidemics in the 2007-2008 influenza season . 9
N Peak timing by population for all counties with epidemics in the 2008-2009 influenza season . 10
O Summary of onset timing as a function of the cumulative proportion of the population in the influenza season . . . 11
P Epidemic duration by spatial scale . . . 12
Q County-level maps of disease burden and spatial aggregation difference for an example influenza season (2006-2007) . . . 13
R Peak intensity spatial aggregation difference between state and county values across all seasons 14
S Onset timing spatial aggregation difference between state and county values across all seasons 14
T Scatterplot of spatial aggregation difference and within-state variance in onset timing by influenza season . . . 15
U Scatterplot of spatial aggregation difference and within-state variance in peak season timing by influenza season . . . 16
V Scatterplot of spatial aggregation difference and within-state variance in early season intensity by influenza season . . . 17
W Scatterplot of spatial aggregation difference and within-state variance in peak season intensity by influenza season . . . 18

Supplementary Tables

A Comparison of region-county spatial aggregation differences between onset and peak season measures . . . 13
B Comparison of region-county and state-county differences for four measures of disease burden 13

S1 Supplementary Methods

The spatial aggregation differences have spatial dependence and temporal structure with multiple observations per county, thus violating the assumptions of the classical paired t-test. Consequently, comparisons of spatial aggregation difference were assessed with Bayesian intercept models (effectively, a Bayesian paired t-test for spatially correlated data) that accounted for county spatial dependence (See SM).

$$\delta_i = \beta_0 + \phi_i \tag{1}$$

where δ_i is defined as the difference between two sets of spatial aggregation differences (e.g., peak versus onset timing, peak versus onset intensity, or region-county versus state-county for a single measure) for county i in a given influenza season. We modeled county spatial dependence ϕ_i with an intrinsic conditional autoregressive (ICAR) model, which smooths model predictions by borrowing information from neighbors [33]:

$$\phi_i | \boldsymbol{\phi}_{j,-i}, \tau_\phi \sim \text{Normal}\left(\frac{1}{\xi_i} \sum_{i \sim j} \phi_j, \frac{1}{\xi_i \tau_\phi}\right), \tag{2}$$

where ξ_i represents the number of neighbors for node i, $\boldsymbol{\phi_{j,-i}}$ represents the neighborhood of node i, which is composed of neighboring nodes j (neighbors denoted $i \sim j$). The precision parameter is τ_ϕ (Equation 2).

The results of this ICAR model were compared to that of an iid error model, where the term ϕ_i is replaced with independent and identically distributed error terms for each node i. We chose the ICAR model over the iid error model for all tests after reviewing the model fits and the Deviance Information Criterion for the two sets of models.

The intercept model was implemented with approximate Bayesian inference in R using Integrated Nested Laplace Approximations (INLA) with the `INLA` package (www.r-inla.org) in order to facilitate the spatial dependence error term [23,24]. If the 95% credible intervals for β_0 fail to overlap with zero, we interpret that there is a statistically significant difference between the measures contributing to δ_i. We used relatively non-informative normal priors for β_0 and relatively non-informative log-gamma priors for the precision term τ_ϕ.

S2 Supplementary Results

S2.1 County population has no consistent association with onset timing

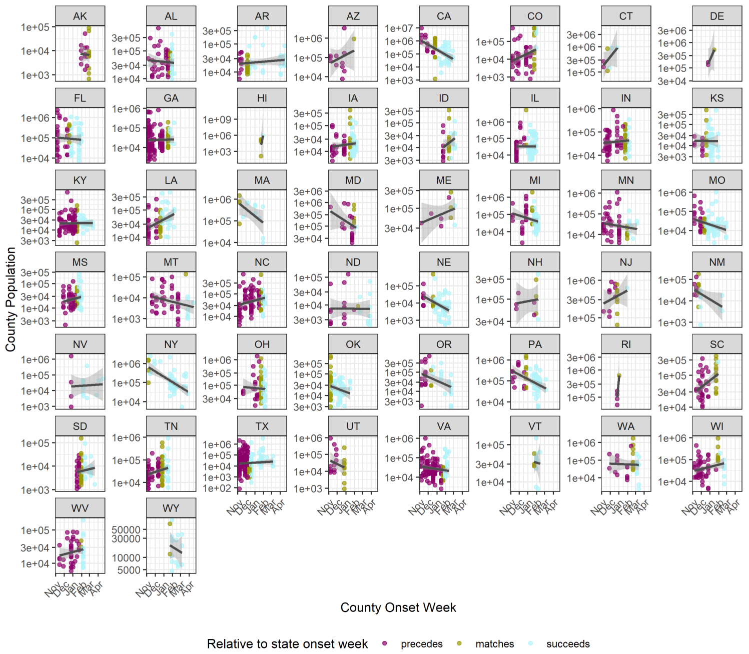

Fig A: **Onset timing by population for all counties with epidemics in the 2002-2003 influenza season.** County points are shaded according to whether onset timing precedes (purple), matches (green), or succeeds (blue) state onset timing in that state.

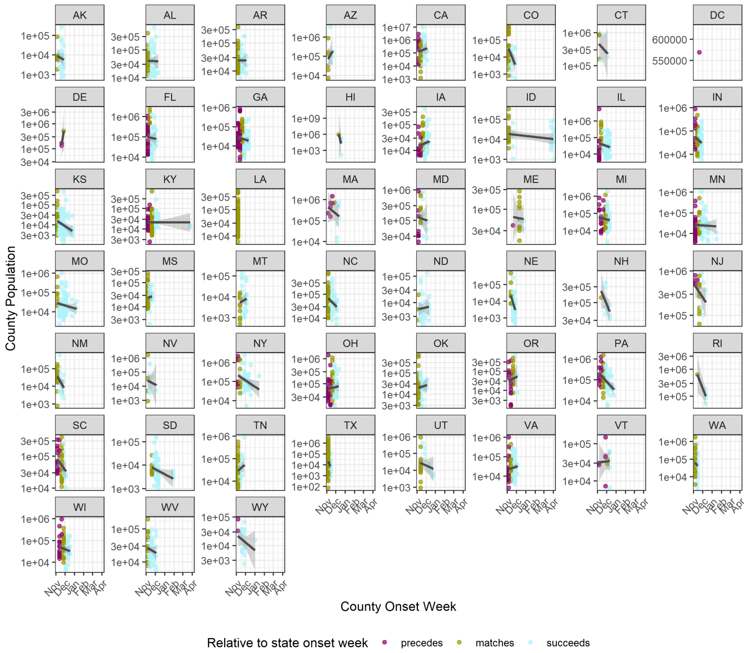

Fig B: **Onset timing by population for all counties with epidemics in the 2003-2004 influenza season.** County points are shaded according to whether onset timing precedes (purple), matches (green), or succeeds (blue) state onset timing in that state.

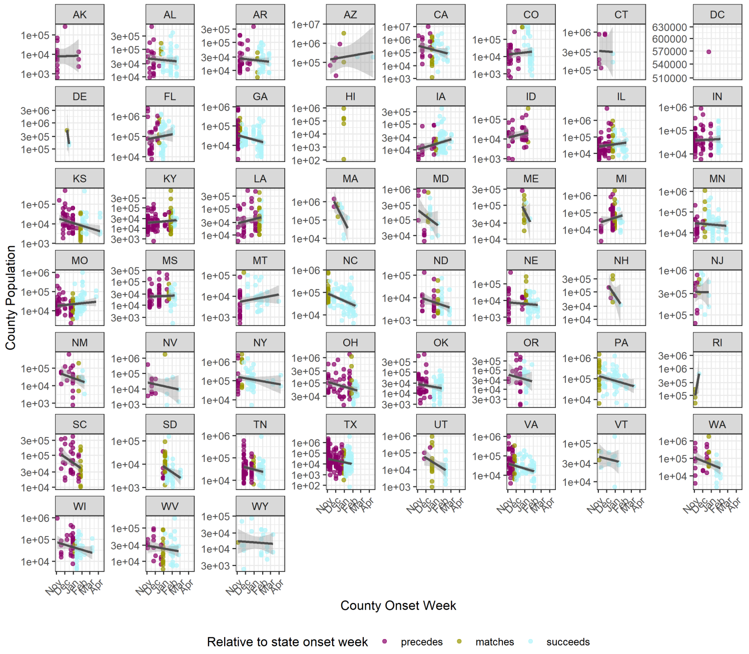

Fig C: **Onset timing by population for all counties with epidemics in the 2004-2005 influenza season.** County points are shaded according to whether onset timing precedes (purple), matches (green), or succeeds (blue) state onset timing in that state.

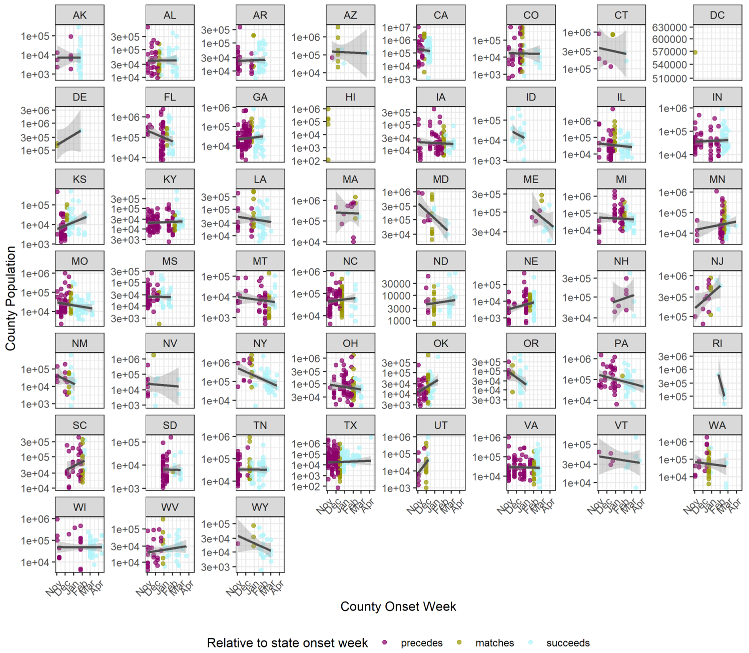

Fig D: **Onset timing by population for all counties with epidemics in the 2005-2006 influenza season.** County points are shaded according to whether onset timing precedes (purple), matches (green), or succeeds (blue) state onset timing in that state.

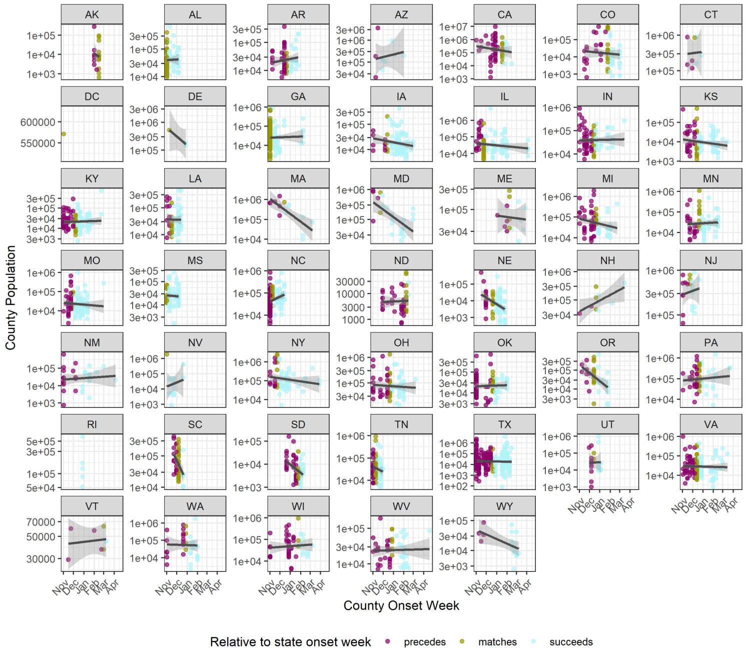

Fig E: **Onset timing by population for all counties with epidemics in the 2006-2007 influenza season.** County points are shaded according to whether onset timing precedes (purple), matches (green), or succeeds (blue) state onset timing in that state.

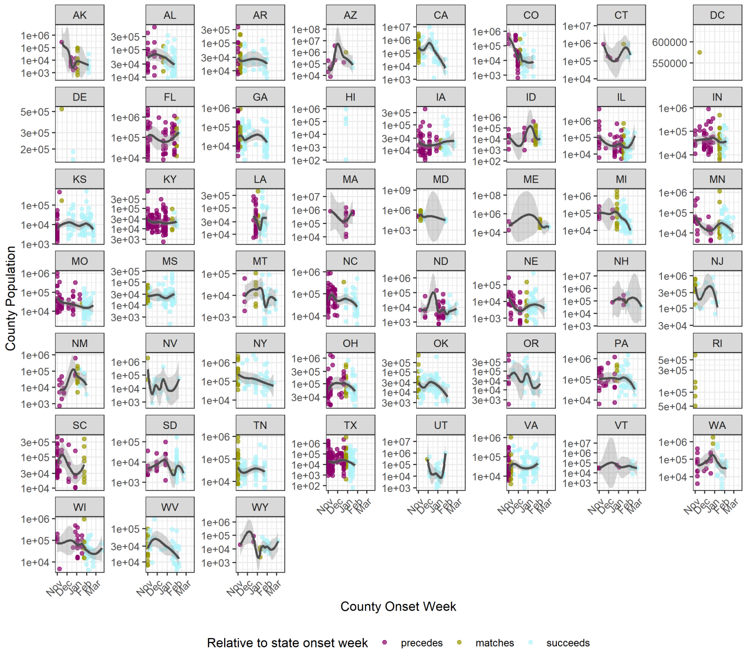

Fig F: **Onset timing by population for all counties with epidemics in the 2007-2008 influenza season.** County points are shaded according to whether onset timing precedes (purple), matches (green), or succeeds (blue) state onset timing in that state.

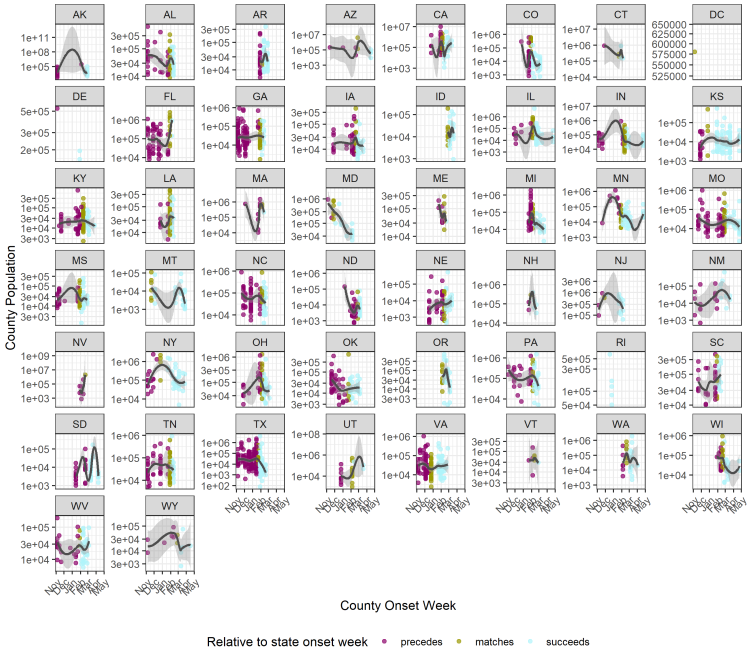

Fig G: **Onset timing by population for all counties with epidemics in the 2008-2009 influenza season.** County points are shaded according to whether onset timing precedes (purple), matches (green), or succeeds (blue) state onset timing in that state.

S2.2 County population has no consistent association with peak timing

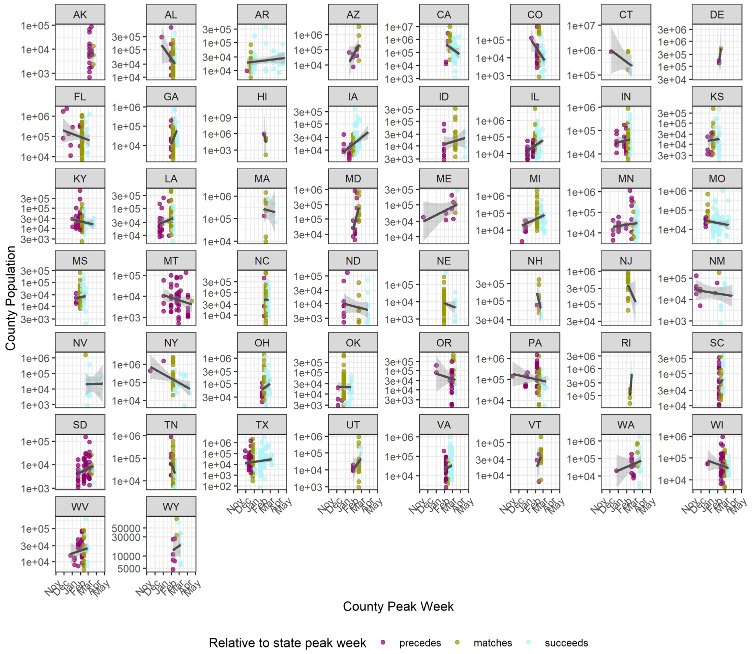

Fig H: **Peak timing by population for all counties with epidemics in the 2002-2003 influenza season.** County points are shaded according to whether peak timing precedes (purple), matches (green), or succeeds (blue) state peak timing in that state.

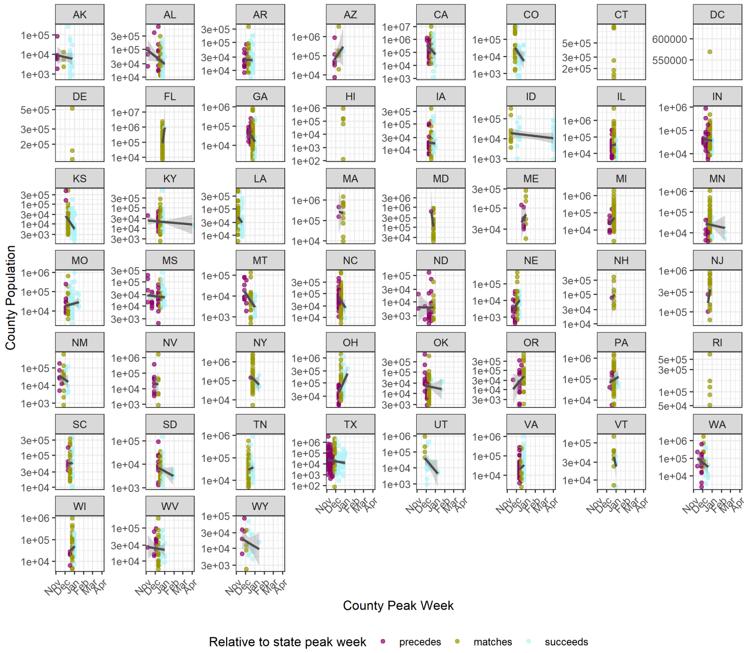

Fig I: **Peak timing by population for all counties with epidemics in the 2003-2004 influenza season.** County points are shaded according to whether peak timing precedes (purple), matches (green), or succeeds (blue) state peak timing in that state.

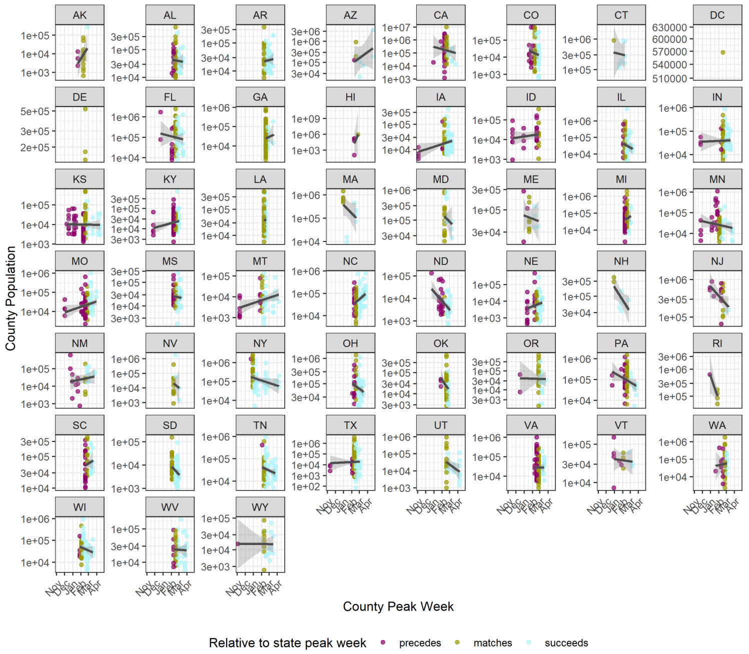

Fig J: **Peak timing by population for all counties with epidemics in the 2004-2005 influenza season.** County points are shaded according to whether peak timing precedes (purple), matches (green), or succeeds (blue) state peak timing in that state.

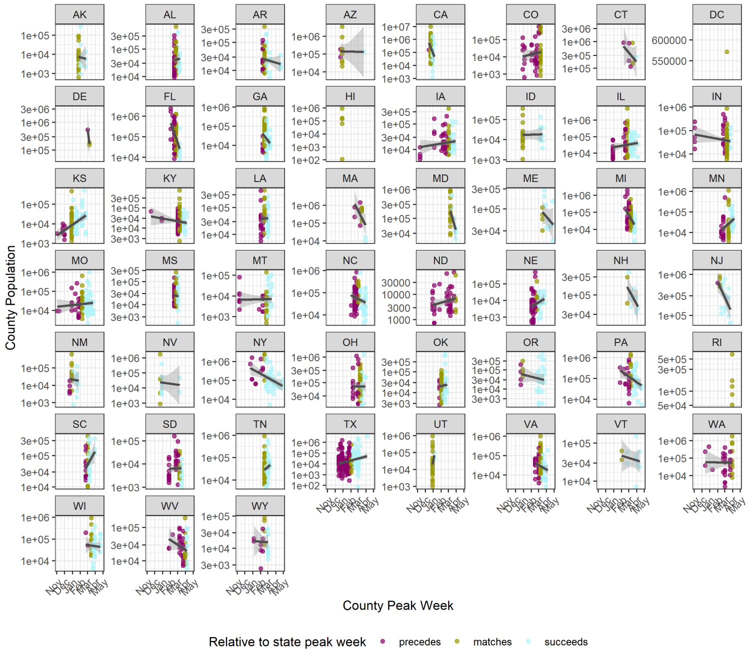

Fig K: **Peak timing by population for all counties with epidemics in the 2005-2006 influenza season.** County points are shaded according to whether peak timing precedes (purple), matches (green), or succeeds (blue) state peak timing in that state.

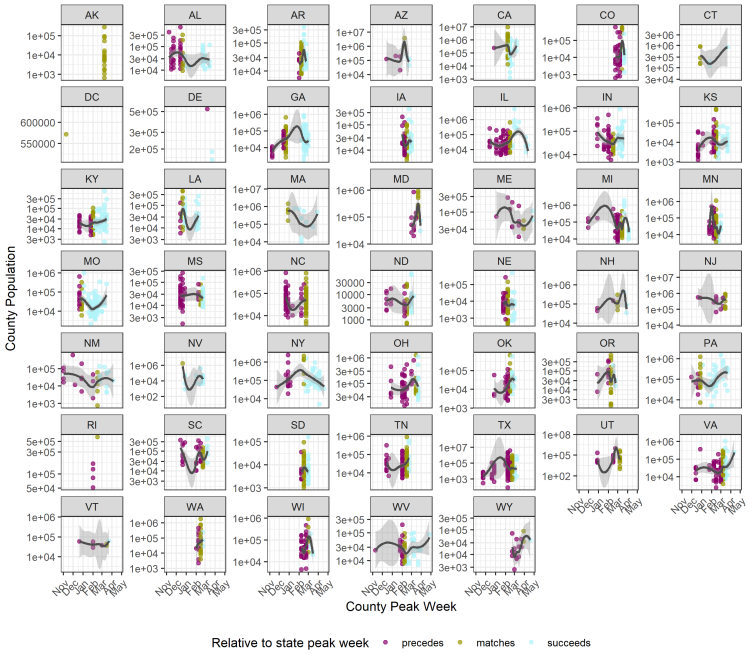

Fig L: **Peak timing by population for all counties with epidemics in the 2006-2007 influenza season.** County points are shaded according to whether peak timing precedes (purple), matches (green), or succeeds (blue) state peak timing in that state.

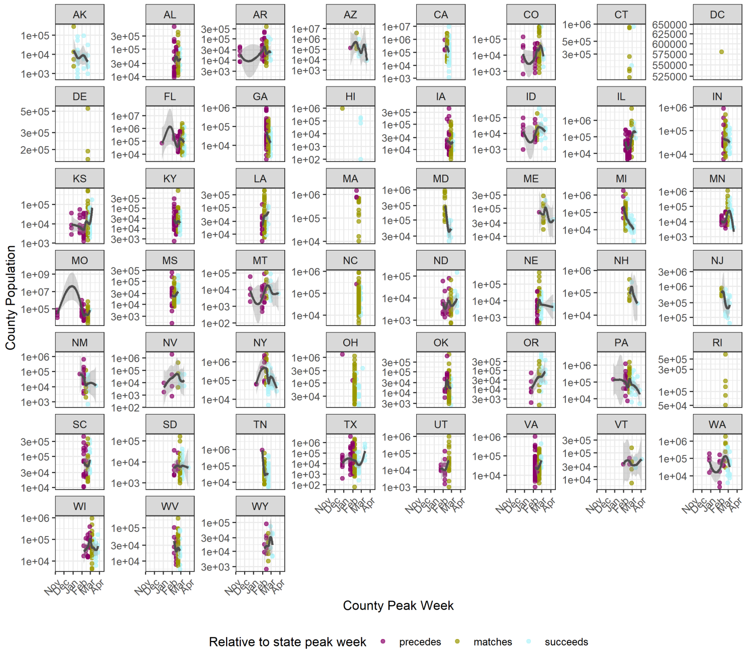

Fig M: **Peak timing by population for all counties with epidemics in the 2007-2008 influenza season.** County points are shaded according to whether peak timing precedes (purple), matches (green), or succeeds (blue) state peak timing in that state.

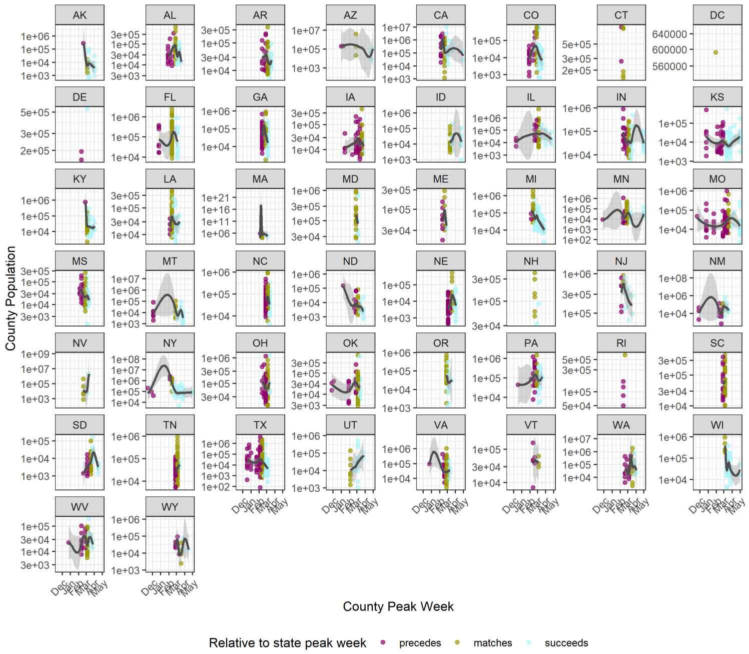

Fig N: **Peak timing by population for all counties with epidemics in the 2008-2009 influenza season.** County points are shaded according to whether peak timing precedes (purple), matches (green), or succeeds (blue) state peak timing in that state.

S2.3 Influenza season features across spatial scales

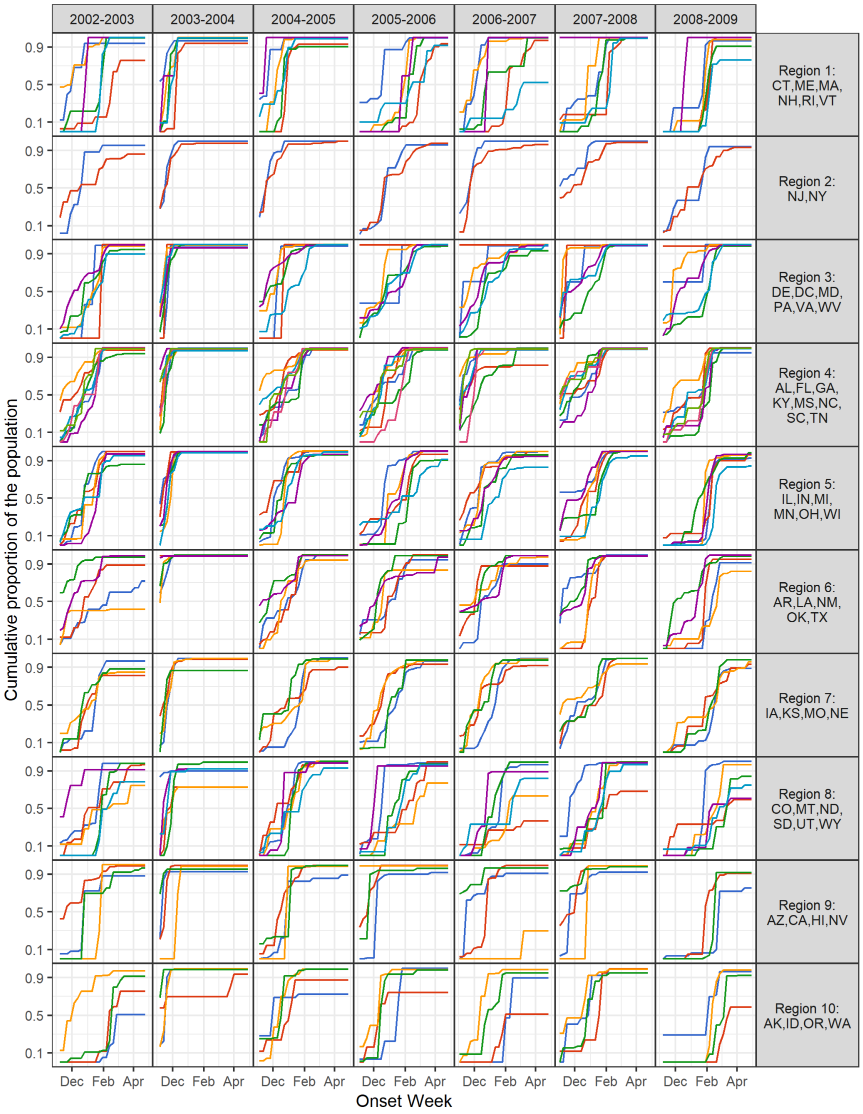

Fig O: **Summary of onset timing as a function of the cumulative proportion of the population in the influenza season.** We present the cumulative percentage of county populations that have experienced influenza season onset over weeks during the influenza season. Each colored line represents a single state and data for all seasons (columns) are shown grouped by HHS region (rows). Not all counties experience influenza seasons every year, some of the cumulative population proportions remain below one even at the end of the potential influenza season period.

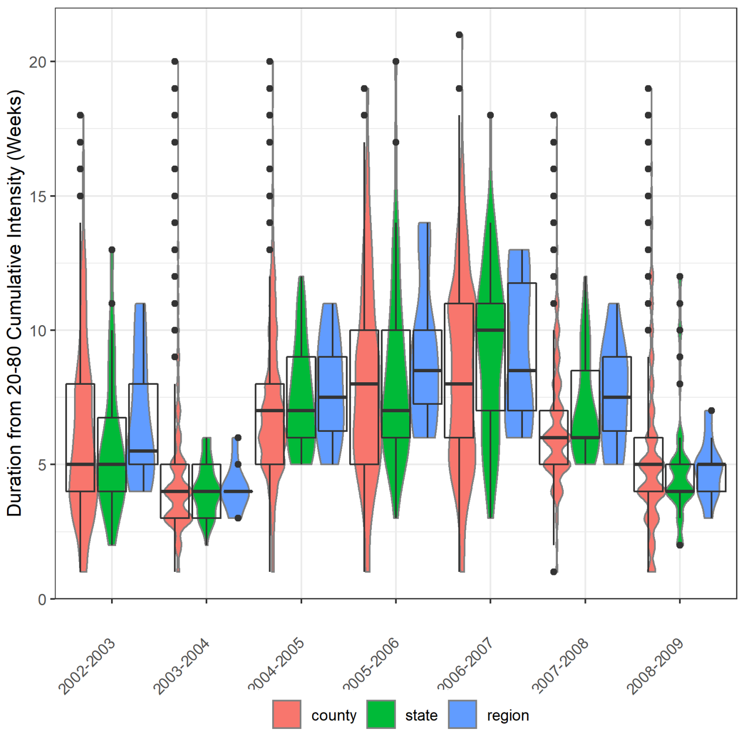

Fig P: **Epidemic duration by spatial scale.** Epidemic duration was defined as the number of weeks between achieving the 20% and 80% cumulative intensity.

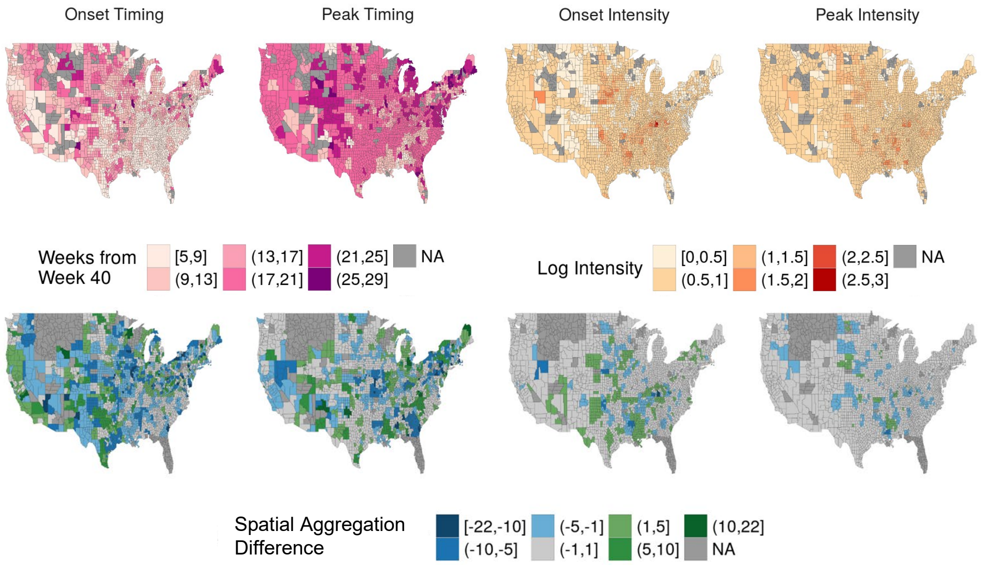

Fig Q: **County-level maps of disease burden and spatial aggregation difference for an example influenza season (2006-2007).** We present county-level disease burden for onset timing, peak timing, onset intensity, and peak intensity (top row) and their associated state-county spatial aggregation differences (bottom row). Timing measures are reported in number of weeks from week 40 while magnitude measures are reported as log intensity. Here, spatial aggregation difference represents the difference between state and county values of disease burden, where negative values (blue) indicate that state-level data underestimated intensity or had earlier timing than county-level data, and vice versa. The map base layer is from the US Census Bureau.

Table A: **Comparison of region-county spatial aggregation differences between onset and peak season measures.** Negative estimates indicate that peak timing had smaller differences than onset timing or that peak intensity had smaller differences than onset intensity. Bolded values denote mean estimates that were statistically significant, where the 95% credible intervals did not overlap with zero.

**Region-County**	**Estimate (95%CI)**
Peak-Onset Timing	**0.07** (0.01, 0.14)
Peak-Early Intensity	**-0.48** (-0.50, -0.47)

Table B: **Comparison of region-county and state-county differences for four measures of disease burden**. Positive estimates indicate that region-county differences are greater than state-county differences, and vice versa. Bolded values denote mean estimates that were statistically significant, where the 95% credible intervals did not overlap with zero.

**Comparison**	**Estimate (95%CI)**
Onset timing	**0.04** (3.9E-4, 0.07)
Peak timing	**0.31** (0.28, 0.34)
Onset intensity	**-0.03** (-0.04, -0.02)
Peak intensity	**-0.20** (-0.20, -0.19)

S2.4 Operational implications of spatial aggregation difference

Peak intensity had consistently low variability in spatial aggregation difference across seasons (Fig R). Onset timing had consistently high variability in spatial aggregation difference across seasons (Fig S).

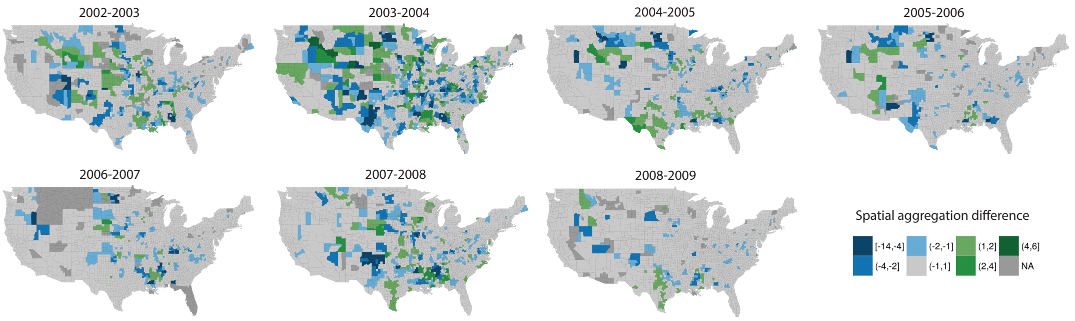

Fig R: **Peak intensity spatial aggregation difference between state and county values across all seasons.** Here, spatial aggregation difference represents the difference between state and county values of disease burden, where negative values (blue) indicate that state-level data underestimated intensity than county-level data, and vice versa. The map base layer is from the US Census Bureau.

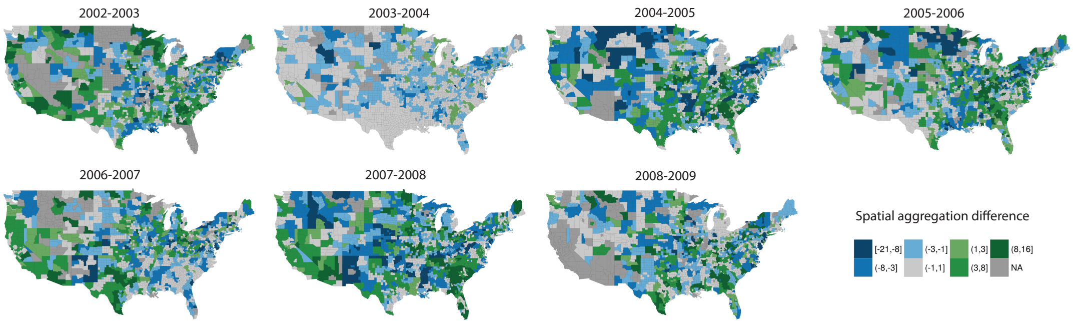

Fig S: **Onset timing spatial aggregation difference between state and county values across all seasons.** Here, spatial aggregation difference represents the difference between state and county values of disease burden, where negative values (blue) indicate that state-level data had earlier timing than county-level data, and vice versa. The map base layer is from the US Census Bureau.

S2.5 Heterogeneity is associated with greater spatial aggregation differences

We hypothesized that spatial aggregation difference was positively associated with spatial heterogeneity in the disease burden measure of interest. For example, as the county-level variation in peak intensity increases within a given state, we might expect that a state-level estimate of peak intensity would have greater absolute difference. We note that spatial aggregation differences for counties in a given state will not sum to zero; as would be done in public health departments, we process our county and state time series directly from the raw surveillance counts and ILI baselines and epidemic periods are identified independently.

To examine the relation between within-state variation and spatial aggregation difference, we examined the Pearson correlation coefficient between the within-state variance in disease burden and the absolute magnitude of state-county error for each measure and flu season in our study period (28 total comparisons). We found statistically significant positive correlations ranging from 0.34 to 0.95 (p-value less than 0.05) for all but four onset measure comparisons —onset timing for the 2005-06, 2006-07, and 2007-08 seasons and onset intensity for the 2006-07 season (Fig T, Fig U, Fig V, Fig W). While some results may be unduly influenced by outlier points, we note that the overall pattern indicates a relationship between variation and error.

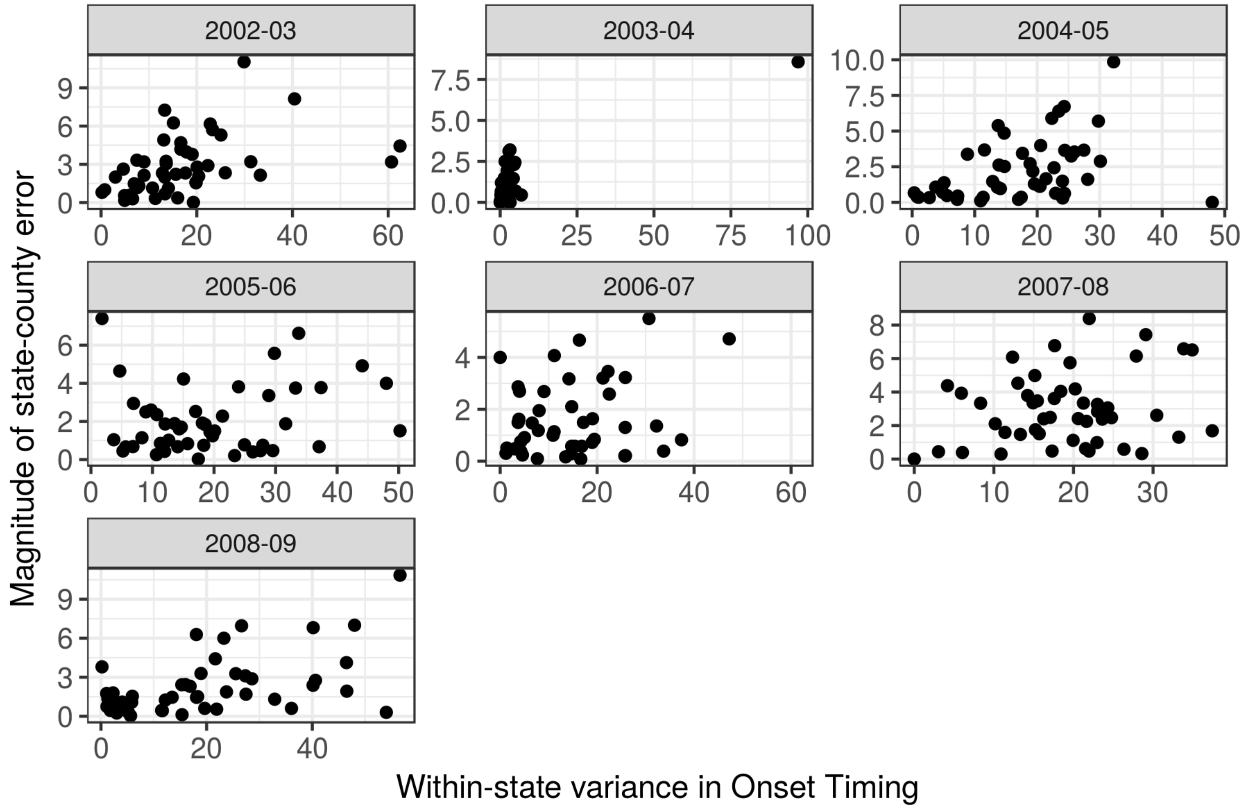

Fig T: **Scatterplot of spatial aggregation difference and within-state variance in onset timing by influenza season.**

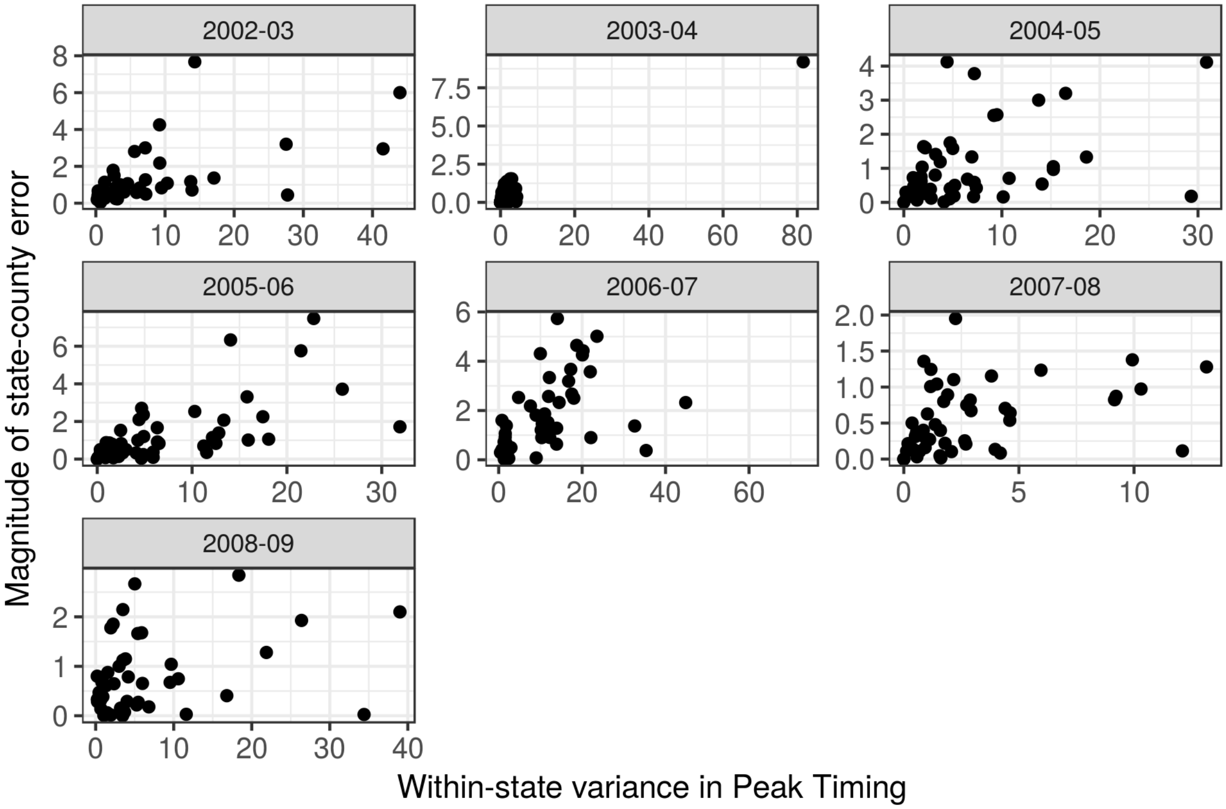

Fig U: **Scatterplot of spatial aggregation difference and within-state variance in peak season timing by influenza season.**

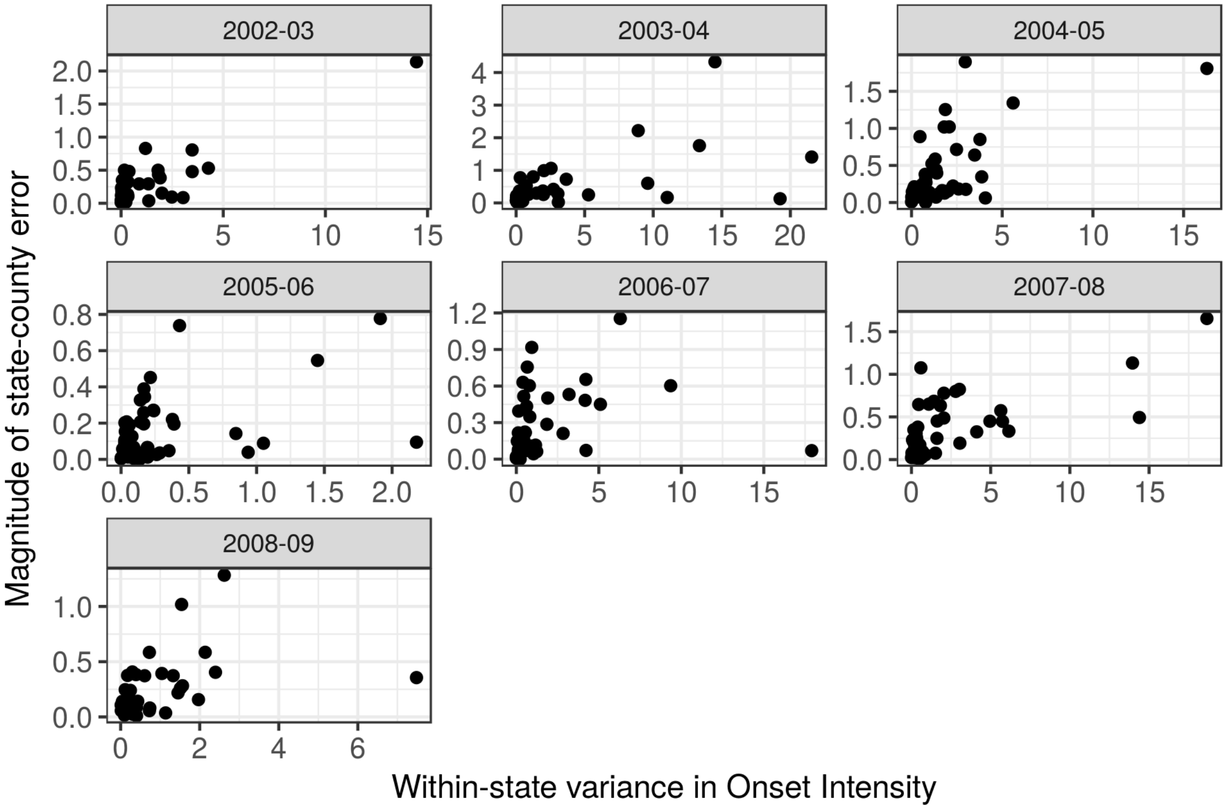

Fig V: **Scatterplot of spatial aggregation difference and within-state variance in early season intensity by influenza season.**

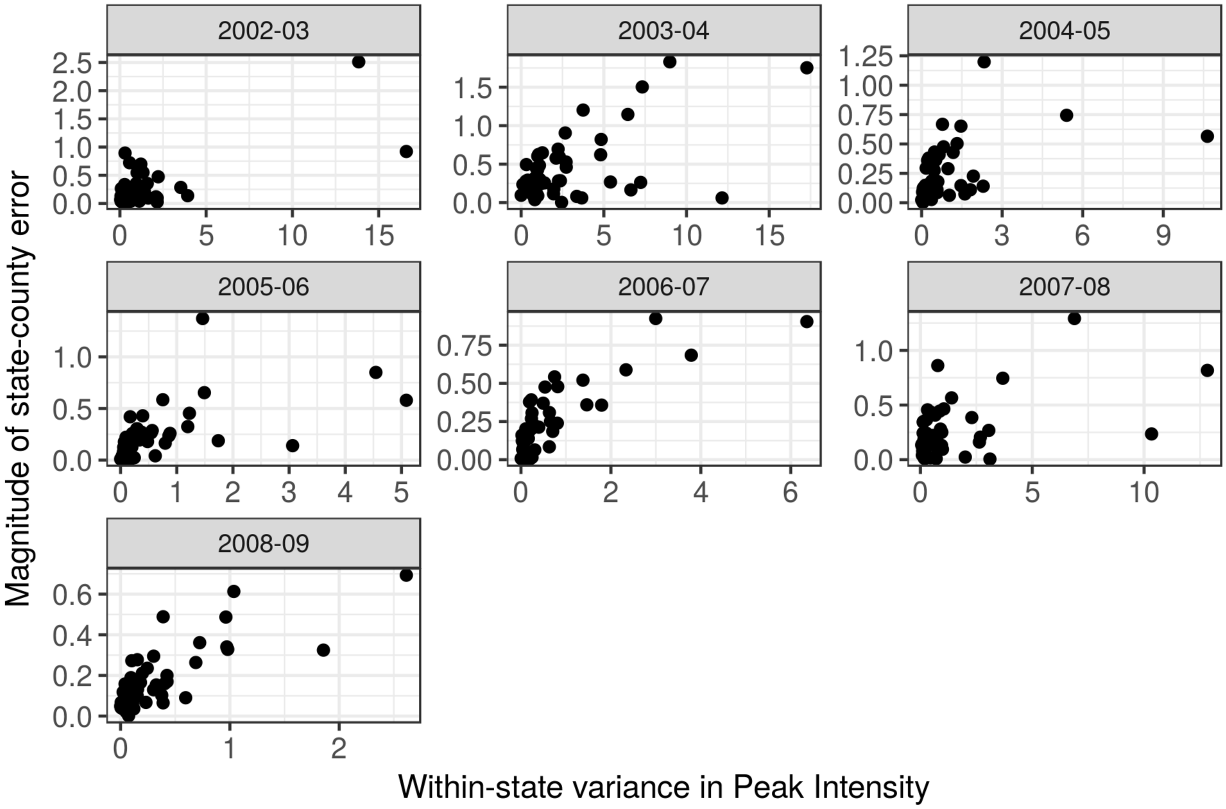

Fig W: **Scatterplot of spatial aggregation difference and within-state variance in peak season intensity by influenza season.**
