# Supplementary material for: Spatial aggregation choice in the era of digital and administrative surveillance data
Source: PLOS Digit Health. 2022 Jun 3;1(6):e0000039. doi: 10.1371/journal.pdig.0000039 (PMC9931313; doi:10.1371/journal.pdig.0000039)
Supplement: Attachment — Submitted filename: Scales MS rebuttal 1.docx [file pdig.0000039.s002.docx]

# Journal Requirements:

1. Please update your Competing Interests statement. If you have no competing interests to declare, please state: “The authors have declared that no competing interests exist.”

**Done.**

2. We ask that a manuscript source file is provided at Revision. Please upload your manuscript file as a .doc, .docx, .rtf or .tex. If you are providing a .tex file, please upload it under the item type ‘LaTeX Source File’ and leave your .pdf version as the item type ‘Manuscript’.

**Done.**

3. Please provide separate figure files in .tif or .eps format only and remove any figures embedded in your manuscript file. Please ensure that all files are under our size limit of 20MB.

For more information about how to convert your figure files please see our guidelines: <https://nam02.safelinks.protection.outlook.com/?url=https%3A%2F%2Fjournals.plos.org%2Fdigitalhealth%2Fs%2Ffigures&amp;data=04%7C01%7Celizabeth.c.lee%40jhu.edu%7C5609b84fc4ba4eba973408d9e289ae57%7C9fa4f438b1e6473b803f86f8aedf0dec%7C0%7C0%7C637789903234706821%7CUnknown%7CTWFpbGZsb3d8eyJWIjoiMC4wLjAwMDAiLCJQIjoiV2luMzIiLCJBTiI6Ik1haWwiLCJXVCI6Mn0%3D%7C3000&amp;sdata=SQv4w6bH4usknE3AnI6Hfv%2FvqcAm8AUnt7YZhnrVFS8%3D&amp;reserved=0>

**Done.**

4. Please provide us with a direct link to the base layer of the map used in Figure 2, Figure S17, Figure S18, Figure S19, and ensure this location is also included in the figure legend.

Please note that, because all PLOS articles are published under a CC BY license (creativecommons.org/licenses/by/4.0/), we cannot publish proprietary maps such as Google Maps, Mapquest or other copyrighted maps. If your map was obtained from a copyrighted source please amend the figure so that the base map used is from an openly available source.

Please note that only the following CC BY licences are compatible with PLOS licence: CC BY 4.0, CC BY 2.0 and CC BY 3.0, meanwhile such licences as CC BY-ND 3.0 and others are not compatible due to additional restrictions. If you are unsure whether you can use a map or not, please do reach out and we will be able to help you.

**The base layers for Figures 2 and S17-19 come from the US Census Bureau and are in the public domain. US Census Bureau shapefiles may be found here:** [**Cartographic Boundary Files (census.gov)**](https://www.census.gov/geographies/mapping-files/time-series/geo/cartographic-boundary.html)**. We added a sentence to each figure caption that says: “The map base layer is from the US Census Bureau.”**

5. Please review your reference list to ensure that it is complete and correct. If you have cited papers that have been retracted, please include the rationale for doing so in the manuscript text, or remove these references and replace them with relevant current references. Any changes to the reference list should be mentioned in the rebuttal letter that accompanies your revised manuscript. If you need to cite a retracted article, indicate the article's retracted status in the References list and also include a citation and full reference for the retraction notice.

**We updated reference 25 (Rosensteel et al) to show that the article is now in press at AJE and added reference 30 (Luca et al) to support a new sentence in the discussion about the role of travel and winter holidays on influenza diffusion.**

# Additional Editor Comments :

Dear authors, we have received all reviewers' decisions, and please refer to their comments below for the revision. In particular, both Reviewer #1 and #3 suggest better explanations on some definitions and terminology. Please address these feedbacks accordingly. Many thanks for your submission and apologize for the rather long reviewing process.

**Thank you for this feedback on our work. We have modified our methods section in an attempt to improve the clarity of our metrics and terminology. Please see the detailed responses below.**

# Reviewer #1:

This paper describes how spatial aggregation (region, state, and county level) can influence measures of disease onset and peak while using non-traditional surveillance data sources. Using ILI and medical claims data as a use case, this work shows how differences in early season measures are observed across different spatial aggregations. In my opinion, these findings contribute to disease surveillance and epidemiological work, especially regarding non-traditional data sources. To the extent of my knowledge, the methods are appropriate for assessing this research question. The study findings, presentation of the work, and rigor of the methods all contribute to my recommendation to publish with minor revisions.

**Thank you for this positive review of our work.**

The overall paper provides a clear narrative about how spatial aggregation choice may impact epidemiological inference of non-traditional data sources. While there are no major concerns on my part regarding the study, there are some places in the paper that could benefit from refined language clarity, and enrichment. These areas are detailed below.

**Thank you for the suggestions. Please see our specific edits below.**

In the introduction, the wording around “political administrative boundaries” in the first paragraph needs addressing. Perhaps there is a word missing.

**We corrected the typo: “As these data are typically collected at centralized locations like sentinel healthcare facilities and summarized according to political administrative boundaries…”**

The introduction also mentions that “methods that account for the hierarchical nature of spatial data have been developed to improve disease mapping and the study of disease dynamics” ahead of a paragraph that details that this does not necessarily apply to non-traditional disease data. I recommend clarifying the statement to show it is more applicable to traditional epi data, or that this work needs to be done for non-tradition.

**We added more specificity to this sentence to indicate that they do not apply to non-traditional disease data: “In epidemiology, statistical methods that account for the hierarchical nature of spatial data have been developed to improve disease mapping for small area aggregated health data.”**

I also recommend reviewing the text to remove the same descriptive word more than once in a statement, such as often in ““Non-traditional data are more voluminous and often collected at the individual level, but they often capture a convenience sample limited by user biases.”

**We reviewed the text and made edits for conciseness throughout.**

For the methods, I think adding what definition of ILI was used (IMS health definition, CSTE, etc.) would be helpful detail. Elaborating on the definitions of weekly ILI visits and diagnoses would also be helpful. Are these diagnoses of ILI, flu, etc. None of the enrichments need to be lengthy or exhaustive, just more informative.

**We added this sentence: “ILI was defined with International Classification of Diseases, Ninth Revision (ICD-9) codes for: direct mention of influenza, fever combined with respiratory symptoms or febrile viral illness, or prescription of oseltamivir, while any diagnosis visits represent all possible medical diagnoses including ILI (also see \cite{Lee2018}).”**

I am interested in how expected prevalence was calculated with population weights yet did not vary across counties. Some enrichment around this would be interesting, perhaps in the discussion.

**The language in the previous draft was ambiguous; there is only a single, national-level expected prevalence value for a given season and it is calculated as the population-weighted mean of the associated intensity measure. This description now reads:**

**“Proxies for prevalence during the onset flu season and peak flu season were calculated as relative risks; the onset intensity and peak intensity for a given county, state, or region was defined as its risk relative to a single, national `expected' onset and peak prevalence, respectively. This `expected prevalence,' calculated for each influenza season, was the population-weighted mean of the associated intensity measure.”**

Additionally, where the time series of season intensity described in “Inferring probable source location” different than the time series sued to defined intensity? For the Euclidean distances, it is the only measurement in its section that is only county, not state or county. How, if any, where distances state to county handled?

**There was a typo in the previous version of the manuscript. Analyses were performed at the county scale or the state scale, so there is no measurement of state to county distances. The related sentence now reads:**

**“Using seasonal time series of intensity, we identified the top 10\% of locations (at the county or state scale) with the earliest epidemic onset for each season as potential source locations and calculated the Euclidean distances between the centroids of potential source locations and all other locations.”**

Lastly, clearly stating the four county-level summary measures of disease burden after the foist use of this language would be a helpful detail to readers, providing more clarity as they navigate the paper.

**We added clarifying text to the beginning of this subsection, which reads:**

**“The study considered five measures of influenza disease burden – two measures of timing (onset and peak flu season timing), two measures of intensity (onset and peak intensity), and epidemic duration – at county, state, and region scales.”**

In the results, I think you should specify the following is across all observed seasons: “We found disagreement in the top two most probable source states and the top 50 most probable source counties (Figure 2)”.

**We added “Across all seasons,” to the beginning of this sentence.**

In the sub section “A majority of county data streams achieve onset and peak timing milestones before state data streams”, please address the repeating word (achieved) in the sentence beginning with “State-level flu season onset and peak timing tended”.

**Done.**

Additionally, I suggest a slight re-phrase so this does not read exactly like methods section: “We defined spatial aggregation difference as the difference between a give influenza disease burden measure at aggregated spatial scales”.

**Done.**

For Figure 2, the county data for HHS region 1 is more difficult to see than the other HHS regions. Highlighting the difference in scale for intensity across regions in the caption would be informative. While the figure’s focus is intraregional comparison across spatial scale, it might

be good to note the differences.

**We assume the reviewer is referring to Figure 1 here. We have added a sentence to the Figure 1 caption explaining that the relatively small number of counties may explain the lack of apparent variability in Region 1.**

For Table 1, is the text starting with “The two negative estimates indicate” part of the title? Should that be moved to the caption? Or explained in the text describing results in the figure?

**We modified this text so it provided interpretation of the table values:**

**“Negative values mean that spatial aggregation estimates for peak measures were smaller than spatial aggregation differences for onset measures.”**

For the discussion, I would emphasize that the goal of this study is more focused on influence of spatial aggregation, especially as novel data streams become available for influenza. I would include the goals around spatial aggregation in this sentence as it was the focus, rather than the current phrasing.

**We agree with the reviewer’s suggestion. The first two sentences of the discussion now read:**

**“Administrative health records, social media streams like Twitter, and participatory surveillance systems like Influenzanet and Flu Near You are increasingly available for influenza surveillance, but use of these data for epidemiological analysis is subject to `spatial aggregation choice' \cite{Bansal2016, Lee2016}. In this study, we examined how an arbitrary choice in the spatial aggregation of non-traditional disease data sources may influence estimates of disease burden and epidemiological understanding of an outbreak.”**

Some additional considerations to enrich this section include additional biases specific to medical claims data that could relate to the results seen in spatial aggregation (beyond geographic coverage). This could include access to care, facility aggregation (particularly in rural areas), etc. Similarly, it may be interesting to briefly address how mobility may play into differential results of spatial aggregation in early and peak seasons. Lastly, detailing why onset and peak location and timing are important to capture arcuately would be a good addition.

**Thank you for the suggestions. We added more examples in the sentences about data reporting biases. This now reads:**

**“Our conclusions about when and how spatial aggregation choice is most important may be conflated with other data reporting processes, such as the expanding geographic coverage of our medical claims data over time, the distribution of reporting healthcare facilities, variability in reporting quality (driven by differences in healthcare and surveillance resources), clustered use of certain ICD-9 diagnosis codes (driven by hospital practices or knowledge-sharing between physicians, for example), and healthcare access, as well as the stochastic variation in influenza season dynamics itself. In addition, changes of mobility over time (e.g. due to family or touristic travels during the winter holidays) associated with changes of contact patterns (e.g. due to closure of schools during holidays) are known to contribute to influenza diffusion \cite{Ewing2016,Luca2018} and may therefore affect the spatial aggregation.”**

**We also added more to describe the policy relevance of the timing and intensity measures we considered in paragraph 4 of the discussion:**

**“Delayed detection of season onset and inaccurate estimation of early season intensity may lessen agility of policymakers and healthcare facilities to anticipate staffing and hospital supply needs as they prepare for the peak influenza season activity.”**

The findings of this study, especially about the plurality of state populations experiencing ILI onset ahead of state-level data indicating such, are very interesting. I think Figure 2 did a nice job of displaying the onset location differences across county and state level data. ILI is a good use case for this work and it is helpful to see how non-traditional and digital data may be influenced differently by spatial choice than traditional epi data, particularly as more of these data for respiratory illnesses emerge in light of COVID-19.

**Thank you for this positive interest in our work. We appreciate the constructive feedback!**

# Reviewer #2:

Because this manuscript is about the spatial autocorrelation, I think it should be also showed the cold-spot, not only the hot-spot areas. It can be really helpful for the readers and also the policy makers in order to find the comparison between the areas. Also, it can boots the improvement in this research in the future. Perhaps using multivariate approach, in order to find the major or other causes of diseases spread.

**We appreciate the reviewer’s suggestion, but believe that explicit examination of coldspots is outside the scope of this work. The purpose of the study is not just to identify high ILI burden areas, but to show that the choice of spatial scale with non-traditional disease data can have an effect on the perceived disease burden. We considered measures of intensity and timing as we believed these were of greatest interest to policymakers who may want to understand when to anticipate increased needs in hospital staff, beds, and ventilators.**

**We added more to emphasize the influence of spatial aggregation on estimates of burden in the first two sentences of the discussion, which now read:**

**“Administrative health records, social media streams like Twitter, and participatory surveillance systems like Influenzanet and Flu Near You are increasingly available for influenza surveillance, but use of these data for epidemiological analysis is subject to `spatial aggregation choice' \cite{Bansal2016, Lee2016}. In this study, we examined how an arbitrary choice in the spatial aggregation of non-traditional disease data sources may influence estimates of disease burden and epidemiological understanding of an outbreak.”**

**We also added more to describe the policy relevance of the timing and intensity measures we considered in paragraph 4 of the discussion:**

**“Delayed detection of season onset and inaccurate estimation of early season intensity may lessen agility of policymakers and healthcare facilities to anticipate staffing and hospital supply needs as they prepare for the peak influenza season activity.”**

I think it will be really important in order to boost the similar research in other countries, especially in my country, Indonesia, in order to boost the surveillance ILI data and spatial utilization in public health sector.

**Thank you for the interest in applying our research.**

In page 7, I found a technical issue in the paragraph

**It appears that the details of this comment were cut off, so we are unable to address it.**

I think the authors should explain the limitation of the study. In manuscript, it is not explained well for me.

**We added more examples on the limitations of our results on spatial aggregation in paragraph 4 of the discussion. This reads:**

**“Our conclusions about when and how spatial aggregation choice is most important may be conflated with other data reporting processes, such as the expanding geographic coverage of our medical claims data over time, the distribution of reporting healthcare facilities, variability in reporting quality (driven by differences in healthcare and surveillance resources), clustered use of certain ICD-9 diagnosis codes (driven by hospital practices or knowledge-sharing between physicians, for example), and healthcare access, as well as the stochastic variation in influenza season dynamics itself. In addition, changes of mobility over time (e.g. due to family or touristic travels during the winter holidays) associated with changes of contact patterns (e.g. due to closure of schools during holidays) are known to contribute to influenza diffusion \cite{Ewing2016,Luca2018} and may therefore affect the spatial aggregation.”**

# Reviewer #3:

The study makes good contribution to the current knowledge. Spatial aggregation differences are critical to statistical inference out of multi-level data. However, the measurement of some key variables in the study looks not easy to follow. For example, " the onset intensity and peak intensity metrics were defined as risks relative to the ‘expected’ onset and peak prevalence...". It would be difficult for readers to learn how the intensity was exactly measured. What are the "risks"? How "expected" was defined?

**We modified the section on metrics to add more clarifying details and refer the readers to our prior study which details the step-by-step algorithm used to generate intensity measures. The text now reads:**

**“The study considered five measures of influenza disease burden – two measures of timing (onset and peak flu season timing), two measures of intensity (onset and peak intensity), and epidemic duration – at county, state, and region scales. In the below definitions, the intensity of influenza activity in a given location and time refers to the time series of the detrended ILI ratio (See details about the intensity calculation at \cite{**[**Lee2018**](https://journals.plos.org/ploscompbiol/article?id=10.1371/journal.pcbi.1006020)**}).**

**“We defined onset timing as the number of weeks from week number 40 (first week of October) until the first week in the epidemic period. We defined peak timing as the number of weeks from week 40 until the week with the maximum epidemic intensity during the epidemic period. The epidemic duration was the number of weeks where the ILI intensity exceeded the epidemic threshold.**

**“Proxies for prevalence during the onset flu season and peak flu season were calculated like relative risks; the onset intensity and peak intensity for a given county, state, or region was defined as its risk relative to a single, national `expected' onset and peak prevalence, respectively. This `expected prevalence,' calculated for each influenza season, was the county population-weighted mean of the associated intensity measure. The onset flu season was identified as a 2-3 week flu season period with the greatest exponential growth rate, while the peak flu season was identified as the week with the maximum ILI intensity.”**

AND the key variable in this study, spatial aggregation difference, was defined as "the difference between a given influenza disease burden measure...." It was just one sentence measuring this. The authors should elaborate more with this measurement. And they should make it easy to read. How burden is related to spatial aggregation difference?

**Spatial aggregation difference is calculated with subtraction between a burden metric at two spatial scales. Spatial aggregation difference was calculated with subtraction for each burden measure and for different pairs of spatial scales throughout the paper. The section now reads:**

**“We defined spatial aggregation difference as the difference between a given influenza disease burden measure at an aggregated spatial scale (i.e., state or region) and the county spatial scale (e.g., $\mu_{state} - \mu_{county}$, where $\mu$ is a burden measure). As burden measures are normalized, they may be compared across spatial scales and the scale of the spatial aggregation difference is the same as that of each individual burden measure. A positive spatial aggregation difference indicates that state- or region-level data over-represented disease burden magnitude (onset and peak intensity) or had later epidemic timing (onset or peak timing) relative to county measures. Among timing measures, spatial aggregation error of 20 means that state surveillance data presented epidemic onset or epidemic peak 20 weeks after county surveillance data. Among intensity measures, a spatial aggregation error of -1 means that state surveillance data reported $e^{-1} \approx0.37$ times the risk of county surveillance data.”**
